# Enhancing Medical Students’ Reflective Capacity: Utilizing Reflective Practice Questionnaire as an Action Research Diagnostic Tool

**DOI:** 10.7759/cureus.54531

**Published:** 2024-02-20

**Authors:** Diwakar Dhurandhar, Swanand S Pathak, Tripti Chandrakar, Pooja Bhadoria, Vaibhav P Anjankar, Adarshlata Singh, Jagriti Agrawal

**Affiliations:** 1 Anatomy, Pt. Jawahar Lal Nehru Memorial Medical College, Raipur, IND; 2 Pharmacology, Jawaharlal Nehru Medical College, Datta Meghe Institute of Higher Education and Research, Wardha, IND; 3 Community Medicine, Pt. Jawahar Lal Nehru Memorial Medical College, Raipur, IND; 4 Anatomy, All India Institute of Medical Sciences, Rishikesh, Rishikesh, IND; 5 Anatomy, Jawaharlal Nehru Medical College, Datta Meghe Institute of Higher Education and Research, Wardha, IND; 6 Dermatology, Venereology and Leprosy, Jawaharlal Nehru Medical College, Datta Meghe Institute of Higher Education and Research, Wardha, IND

**Keywords:** metacognition, medical students, action research, reflective practice questionnaire, reflective capacity

## Abstract

Introduction

Reflection is the intentional evaluation of one's beliefs or understanding in consideration of the available evidence. Reflection has been noted to enhance profound learning and critical thinking and is an essential foundation of experiential learning. The Reflective Practice Questionnaire (RPQ) is a valid and reliable tool for assessing reflective capacity among medical students. It assesses not only reflective capacity but also other psychological constructs relevant to reflective practice, such as job satisfaction, confidence while interacting with patients, stress during patient interaction, desire for improvement, and feelings of uncertainty. The use of RPQ as a diagnostic tool for improving reflective capacity among medical students is scarcely available in the literature. Thus, the present study aimed to utilize the RPQ for identifying discrete action points for training and future improvement in reflective capacity.

Materials and methods

The present cross-sectional descriptive study was conducted among 300 medical students of a medical college. To identify the clusters or meaningful subgroups within the study population, cluster analysis was done. Inter-correlation between subscales of RPQ was performed by forming Pearson’s correlation matrix to understand the dynamics between various subscales of RPQ.

Results

Five groups were identified in the cluster analysis: typical (25.6%), reflective (27%), non-reflective (16.7%), unconfident (14%), and overconfident (16.7%). This sets the template for specific action points for each group identified above. Reflective capacity was positively correlated with Desire for Improvement (DfI), General Confidence (CG), Communication Confidence (CC), and Job Satisfaction (JS). It was also positively correlated with negative outcomes such as Uncertainty (Unc) and Stress when Interacting with Patients (SiC).

Conclusion

RPQ can be used as a diagnostic tool in terms of reflective capacity for action research.

## Introduction

Reflection is characterized as delving into experiences to enhance the existing body of knowledge and comprehension [1]. The act of reflection is described as a cognitive process with a predetermined objective, employed for the purpose of comprehending and articulating abstract concepts [2]. Reflection has been documented to enhance profound learning, foster critical thinking, and is an essential cornerstone of experiential learning [3]. The National Medical Commission, which regulates medical education in India, has inculcated reflection as a teaching, learning, and formative assessment tool in curriculum-based medical education.

Various tools have been utilized to assess the reflective capacity of medical students. These include self-administered questionnaires such as the Reflection-in-Learning Questionnaire given by Sobral DT [4], a questionnaire on reflection during medical diagnostic reasoning proposed by Mamede S and Schmidt HG [5,6], and the Groningen Reflection Ability Scale (GRAS) suggested by Aukes LC et al. [7], which is a questionnaire to assess reflective capacity in medical practice.

In addition to the self-reported measures of reflection mentioned earlier, other methods involve qualitatively evaluating written aspects of reflection to gauge an individual's capacity for reflection [8]. Other structured ways to assess reflection include evaluating written responses to brief scenarios [9], videos [10], or semi-structured exercises [11].

In response to the need for an effective assessment tool for measuring reflective capacity in all professions serving the general public, Priddis L and Rogers SL in 2017 [12] designed the Reflective Practice Questionnaire (RPQ) to assess other psychological constructs relevant to reflective practice apart from reflective capacity itself, such as Desire for Improvement (DfI), General Confidence (CG), Communication Confidence (CC), Uncertainty (Unc), Stress Interacting with Clients (SiC), and Job Satisfaction (JS). They subsequently administered and validated the tool among medical students [13]. Available literature regarding the use of RPQ as a diagnostic tool for action research is scarce. Thus, the present study was conducted among medical students with the objective of providing insight into various personality attributes corresponding to subscales of RPQ and thus providing a template to work upon and accomplish the desired Reflective capacity levels so that he can become a good physician of first contact.

## Materials and methods

The present cross-sectional descriptive study was conducted among final-year and internship undergraduate medical students from a medical college in central India. The study cohort was similar to Rogers SL et al. [13], who also administered the RPQ among fourth-year medical students from an American university and utilized it as a diagnostic tool. Students absent on survey days or who did not give informed consent were excluded from the study. Thus, the sampling technique adopted was convenient non-probabilistic sampling.

The study proposal was submitted to the Institutional Ethics Committee, Pt. J.N.M Medical College, Raipur, for approval (IRB# No./MC/Ethics/2023/38). Following approval, students were provided with a detailed explanation of the study's purpose, objectives, and potential benefits, allowing them to make an informed decision about their participation. Additionally, assurances of anonymity and data confidentiality were provided.

After obtaining informed consent, the RPQ, a self-administered survey consisting of 40 items, was introduced. This survey offers a comprehensive means of assessing one's reflective abilities. The questionnaire used in the present study is available online as an additional file to the main manuscript of Rogers SL et al. (https://static-content.springer.com/esm/art%3A10.1186%2Fs12909-019-1481-6/MediaObjects/12909_2019_1481_MOESM1_ESM.docx) [13]. The RPQ includes sub-scales covering a variety of aspects such as the desire for enhancement, self-confidence, stress levels, and job satisfaction, proving to be a reliable tool for assessing reflective capabilities [12,13]. The response scale for each item in the RPQ is rated on a Likert scale ranging from 1 to 6 where the number indicates the degree of agreement with the statement. 1 indicates Not at all and 6 suggest Extremely. The calculation of each sub-scale score involves taking the average of the relevant items to obtain the sub-scale score, except for the 'job satisfaction' sub-scale, where one of the items requires reverse scoring before averaging.

After getting the sub-scale scores for the individual participant, hierarchical agglomerative cluster analysis using the weighted average linkage method and absolute value distance as the dissimilarity measure was performed, similar to Rogers SL et al. [13], to identify clusters or meaningful subgroups within the study population. A dendrogram was also prepared. Based on the classification or cluster to which a student belonged, tailor-made action research plans were devised, setting a template for future research aimed at optimizing students' reflective capacity.

Additionally, inter-correlation between RPQ sub-scales was estimated by forming Pearson’s correlation matrix, similar to Rogers SL et al. [13]. SPSS version 21 (IBM Inc., Chicago) was used for statistical analysis.

## Results

In total, 300 students took part in the survey out of a pool of 330, resulting in a response rate of 90.0% in the current study.

**Figure 1:**
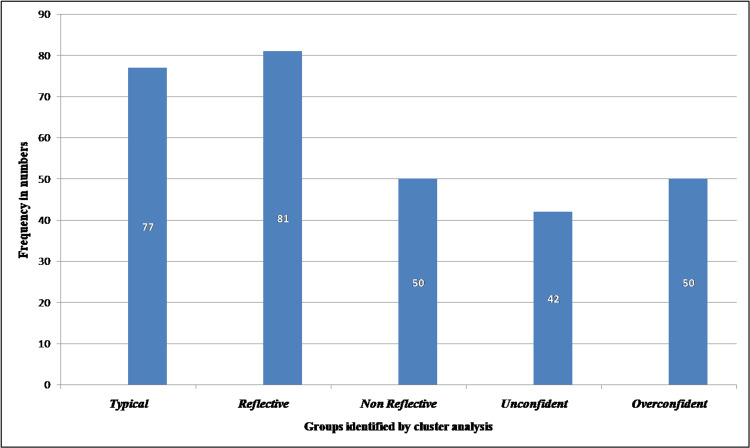
Groups identified by exploratory hierarchical cluster analysis and their frequencies.

Our study revealed five groups or clusters among the study participants by exploratory hierarchical cluster analysis using the weighted average linkage method and absolute value distance as the dissimilarity measure. The number and frequencies of students belonging to each group are shown in Table 1 and Figure 1.

**Table 1:** Various groups identified in cluster analysis: numbers and frequencies. RPQ: Reflective Practice Questionnaire.

Group	Group name	Features	Number (Frequency in %)
1	Typical (T)	Scores on the RPQ are representative of the group's overall statistics	77 (25.6%)
2	Reflective (R)	Individuals with all RPQ subscales at a higher level	81 (27%)
3	Non-reflective (NR)	Individuals with all RPQ subscales at a lower level	50 (16.7%)
4	Unconfident (UC)	Significantly lower general and communication confidence, but with a higher desire for improvement compared to the typical subgroups	42 (14%)
5	Overconfident (OC)	Higher levels of general confidence, communication, and job satisfaction, with lower levels of uncertainty, stress when interacting with patients, and desire for improvement.	50 (16.7%)

These groups were categorized as Typical, Reflective, Non-reflective, Unconfident, and Overconfident.

**Figure 2:**
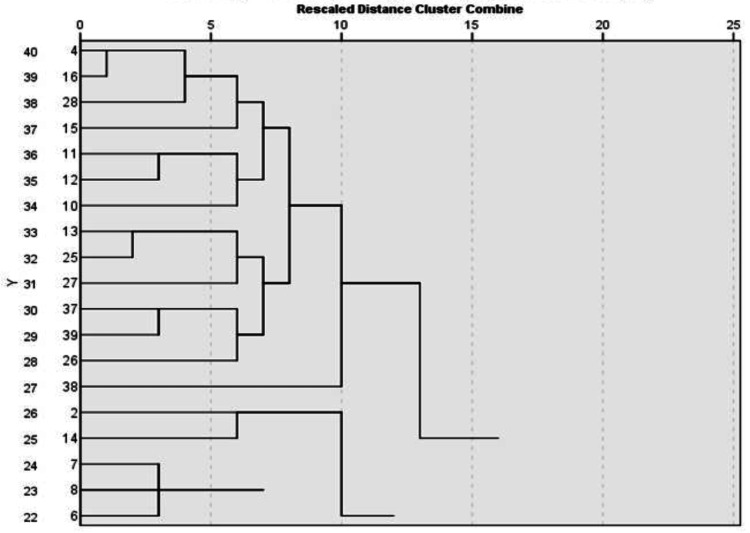
Dendrogram of exploratory cluster analysis of all the participants of the study.

Also, a dendrogram of the exploratory cluster analysis of all the study participants is shown in Figure 2.

In Table 2, inter-correlations among RPQ sub-scales as Pearson’s correlation coefficient are represented.

**Table 2:** Inter-correlations among RPQ sub-scales as Pearson's correlation coefficient. # indicates a p-value of less than 0.01 (2-tailed) - significant correlation between sub-scales. RC: Reflective capacity; DfI: Desire for Improvement; CG: General Confidence; CC: Communication Confidence; Unc: Uncertainty; SiP: Stress when Interacting with Patients; JS: Job Satisfaction.

	RC	DfI	CG	CC	Unc	SiP	JS
RC	1	0.597^#^	0.328^#^	0.582^#^	0.468^#^	0.330^#^	0.636^#^
DfI	0.597^#^	1	-0.023	0.447^#^	0.295^#^	0.049	0.462^#^
CG	0.328^#^	-0.023	1	0.524^#^	-0.031	0.082	0.390^#^
CC	0.582^#^	0.447^#^	0.524^#^	1	0.039	-0.042	0.623^#^
Unc	0.468^#^	0.295^#^	-0.031	0.039	1	0.457^#^	0.179^#^
SiP	0.330^#^	0.049	0.082	-0.042	0.457^#^	1	0.094
JS	0.636^#^	0.462^#^	0.390^#^	0.623^#^	0.179^#^	0.094	1

Reflective Capacity was positively correlated with outcomes such as DfI, CG, CC, and JS. It was also positively correlated with negative outcomes such as Unc and SiC.

## Discussion

The present study evaluates various sub-scales of the RPQ. The RPQ has been suggested as a valid and reliable assessment and evaluation tool [12,13]. In the present study, cluster analysis was performed similarly to Rogers SL et al. [13], who introduced the self-administered RPQ to medical students of an American university and compared the RPQ scores with a cohort of the general public of Australia and another cohort of mental health professionals from Australia who had undergone formal training in reflections. They also validated the RPQ for use among medical students and found this instrument to be reliable [13].

Our study revealed five groups of clusters among study participants by exploratory hierarchical cluster analysis. These groups were categorized as Typical (having RPQ sub-scales values close to the average value obtained in the study), Reflective (having all the RC sub-scales at a higher level), Non-reflective (having all the RC sub-scales at a lower level), Unconfident (significantly lower general confidence and communication but with a higher desire for improvement than the typical sub-groups), and Overconfident (much higher levels of general confidence & communication and job satisfaction with lower levels of uncertainty, stress interacting with patients, and desire for improvement). Our groups differed from those identified by Rogers SL et al. [13], who found the following groups after cluster analysis: Anxious, with substantially high uncertainty and stress interacting with patients (16%); Dissatisfied, with much lower job satisfaction (around 5%); Less Engaged, who expressed less desire for improvement, low job satisfaction, and less confidence than the typical group of students (23%); and Overconfident, with a very high level of confidence in their knowledge, skills, and ability to communicate with patients, low levels of stress and uncertainty, and desire for improvement and RC (7%).

These clusters were identified not to diagnose and earmark the learner into a rigid group but to help them attain the optimal level of reflection and professionalism by receiving feedback according to the findings of the study and providing corrective remedial measures. For non-reflective students, formal reflective writing training such as narrative writing, role plays, and logbooks can be used, and a follow-up assessment can be done after intervention for impact evaluation of the educational intervention. Likewise, for the unconfident group, a training program in communication skills with special reference to doctor-patient interaction can be performed, and an assessment can be done afterward to look for satisfactory improvement.

The inter-correlation between various sub-scales was assessed. It showed that reflective capacity is positively correlated with outcomes such as DfI, CG, CC, and JS. It is also positively correlated with negative outcomes such as Unc and SiC. These are mere correlations and need not have a causal relationship. Our findings are similar to Priddis L and Rogers SL [12], who hypothesized that reflection allows gaining confidence in general and also while interacting with the patient. A person with good reflective capacity would select, plan, and evaluate the experience and thus keep improving their skill sets. A clinician is recognized by the skill sets they carry. Having skills improved in the targeted area, the feeling of confidence within oneself is obvious, and thus, the positive correlation between RC and DfI is self-explanatory. With confidence in patient management and a desire for improvement in clinical acumen, job satisfaction also comes along.

Reflection is a metacognitive activity or thinking about thinking [14]. There is an element of darkness in this process. With too much reflection during or after patient interaction, there may also arise undue anxiousness and accompanying tension. This underlines the positive correlation between RC and SiP sub-scales.

Clara M [15] (2015) highlighted that one tends to reflect in an incoherent situation and augurs for more coherence with reflection. A clinician's life is full of uncertainties where patients with atypical presentations or lesser-known diseases appear. These uncertainties have often been recognized as the heart of a clinician’s practice, which is usually dealt with by all physicians alike. Thus, uncertainty allows a clinician to reflect and rectify performance in similar encounters.

The present study was based on a self-assessment questionnaire. The responder may not have sufficient ability to introspect, thus always giving themselves better scores than what they should have scored. Also, a large number of intrinsic factors like inherent motivation, expectancy, and prior experiences with reflection, and extrinsic factors like the formative and summative nature of assessment, play contextual or confounding roles [16]. Interpretation of the findings of the present study should be done keeping these drawbacks in mind.

Nevertheless, the study can be seen as a starting point for action research with the ultimate aim of aligning the personality attributes of medical students to the roles and behavior as expected by the National Medical Commission in its various vision documents and which is the ideal manner a physician of first contact should interact and conduct with patients. Also, the inter-correlation of various psychometric components of the RPQ helps in generating hypotheses about dynamics between them during doctor-patient interaction.

## Conclusions

In the current study, the RPQ has been employed as a diagnostic instrument to evaluate the levels of sub-scales determining reflective capacity. Cluster analysis and identification of meaningful groups allow different improvement approaches in different groups. The present study sets the template for action research required to bring the subjects their optimal reflective capacity. 
